# Exocytosis, Endocytosis, and Their Coupling in Excitable Cells

**DOI:** 10.3389/fnmol.2017.00109

**Published:** 2017-04-19

**Authors:** Kuo Liang, Lisi Wei, Liangyi Chen

**Affiliations:** ^1^Department of General Surgery, XuanWu Hospital, Capital Medical UniversityBeijing, China; ^2^State Key Laboratory of Membrane Biology, Beijing Key Laboratory of Cardiometabolic Molecular Medicine, Institute of Molecular Medicine, Peking UniversityBeijing, China

**Keywords:** exocytosis, endocytosis, kiss and run, kiss and stay, compound fusion, multivesicular exocytosis, clathrin

## Abstract

Evoked exocytosis in excitable cells is fast and spatially confined and must be followed by coupled endocytosis to enable sustained exocytosis while maintaining the balance of the vesicle pool and the plasma membrane. Various types of exocytosis and endocytosis exist in these excitable cells, as those has been found from different types of experiments conducted in different cell types. Correlating these diversified types of exocytosis and endocytosis is problematic. By providing an outline of different exocytosis and endocytosis processes and possible coupling mechanisms here, we emphasize that the endocytic pathway may be pre-determined at the time the vesicle chooses to fuse with the plasma membrane in one specific mode. Therefore, understanding the early intermediate stages of vesicle exocytosis may be instrumental in exploring the mechanism of tailing endocytosis.

## Introduction

Vesicle exocytosis is a fundamental cellular process that regulates many biological events, such as the release of neurotransmitters, hormones, and cytokines and delivery of proteins and lipids to the plasma membrane for cell repair, growth, migration, and regulation of cell signaling (Alabi and Tsien, 2013; Wu L. G. et al., 2014). In excitable cells, such as neurons and endocrine cells, regulated exocytosis is triggered within milliseconds after membrane depolarization. Upon strong stimulation, a massive fusion of secretory vesicles could occur at designated release sites within a short period of time. Therefore, compared with constitutive exocytosis in non-excitable cells, regulated exocytosis must be equipped with specialized machinery that enables fast, Ca^2+^-dependent, spatially defined exocytosis. Tailing endocytosis must match with exocytosis to recycle exocytosed vesicular components and clear release sites on the plasma membrane in a timely fashion. Based on the kinetics, structures, and molecules involved in different cell types, a variety of exocytosis and endocytosis subtypes have been proposed. However, how these mechanisms are coupled in space and time remains mysterious. Here, we have provided an outline of different exocytic and endocytic processes and how they may be coupled by different factors.

## Exocytosis in excitable cells

Exocytosis requires a merging of the vesicular membrane into the plasma membrane. Through shielding of the negative charge on the bilayer surface, diminishing electrostatic repulsion force, and overcoming the dehydration barrier, two bilayers can merge into one. Formation of an assembled ternary SNARE complex provides the required energy. Depending on the fate of the vesicular components upon lipid merging, exocytosis can progress by fully inter-mixing the vesicular membrane components with the plasma membrane (full fusion), fusing with the plasma membrane via a transient flickering of the fusion pore (kiss and run; An and Zenisek, 2004; Rizzoli and Jahn, 2007; Alabi and Tsien, 2013), or fusing with a partially retained vesicular membrane structure and components at the exocytic site (kiss and stay; Taraska et al., 2003; An and Zenisek, 2004; Tsuboi et al., 2004; Rizzoli and Jahn, 2007).

### Different models of fusion pore formation

Intrinsically, fusion machinery must operate with the formation and the expansion of an omega-shaped pore structure, which minimizes leakage from the vesicle and cytosol during exocytosis. Such a process requires coordinated distortion and controlled disruption of two lipid bilayers to form a water-filling fusion pore, which cannot be observed (van den Bogaart et al., 2010) directly *in vivo* due to its small size and short lifetime (Lindau and Alvarez de Toledo, 2003). Electrophysiological methods, on the other hand, provide a brief glimpse of some pore intermediates. Based on these indirect estimations, there exists three models that describe the fusion pore, a lipidic (Chanturiya et al., 1997) or a proteinaceous (Han et al., 2004) pore or a hybrid of the lipid and protein composition (Bao et al., 2016; Sharma and Lindau, 2016). For a lipidic pore, fusion starts with protrusion of two bilayers toward each other in a very narrow region, followed by the merge of the two proximal monolayers of each bilayer (stalk), the enlargement of the merged region to form one bilayer (hemifusion), and the final formation of a lipidic fusion pore. Formation of the stalk and hemifusion diagram ensures the expansion of the pore without a leak. Such a fusion pore does not require multiple copies of SNARE complexes. Instead, one pair of SNARE proteins, firmly anchored on the vesicular and plasma membrane with transmembrane (TM) segments, may interact with each other to provide the force to pull the different membranes together (van den Bogaart et al., 2010).

In 1987, Almers and co-workers measured the initial pore conductance during exocytosis of mast cells to be 200–300 pS, equivalent to a pore of diameter of ~2 nm (Breckenridge and Almers, 1987). This value is similar to the conductance of K channel, inspiring the early hypothesis that the fusion pore is a proteinaceous gap junction channel. In 2004, using tryptophan scanning mutagenesis of the syntaxin TM anchor, Han et al. identified three critical residues that reduced the amplitude of the foot signal of an amperometry recording (Han et al., 2004). These positions are positioned along one face of the alpha-helix, promoting the idea that they might face the inside of a pore. Based on these results, they proposed a provocative hypothesis that the TM domains of 6–8 syntaxin molecules are arranged in a ring to form one half of a gap-junction-like pore, with the other half formed by the TM domains of synaptobrevin (Syb2). After the formation of the protein-lined pore, the pore could close again (“kiss and run”; Albillos et al., 1997; MacDonald et al., 2006) or allow the membrane lipids to enter to facilitate pore expansion and complete membrane merge (“pore dilation” or “full fusion”). This interpretation is supported by the existence of syntaxin clusters on the plasma membrane of endocrine and synapses (Barg et al., 2010; van den Bogaart et al., 2011), as well as three or more copies of SNARE proteins that are required for the fast release of secretory vesicles (Domanska et al., 2009; Mohrmann et al., 2010). This model, however, requires fusogenic proteins to be in perfect alignment to constrict lipid flow during the initial pore opening, which have not been proved experimentally. Changes in the amplitude of foot signals could be due to different extents of vesicular filling (Sombers et al., 2004) and different dissociation kinetics of neurotransmitters from the vesicular matrix (Reigada et al., 2003). The impact of a syntaxin mutation on the release kinetics thus may provide an alternative explanation. Other amperometric investigations also reported controversial results, such as very large fluctuations of foot signals characteristic of variable and lipidic pores.

A combination of these two models yielded a model with a pore that is both lipidic and proteinaceous. Recently, two Syb2 molecules have been shown to be able to be incorporated within a nanodisc with a diameter of 6 nm, which readily fuses with t-SNARE-containing vesicles and can be blocked by mutations of critical residues in the TM domain of Syb2. Given that such a small nanodisc appeared to be too small to accommodate a lipidic pore, and at least three TM domains are required to line up a proteinaceous pore, these results suggest that the pore itself must be a hybrid of both proteins and lipids (Bao et al., 2016). According to the molecular dynamic simulation, the water-filled fusion pore traversing the membrane and the nanodisc constitutes both the lipid head group and the c-termini of the TM domains of Syb2 and syntaxin (Sharma and Lindau, 2016). Whether such a hybrid model works in real cells remains to be determined.

### Pore opening and full fusion

Under electron microscopy (EM), the earliest seen fusion pores of synaptic vesicles were never <20 nm despite the observed 3–4 ms after a single stimulus (Heuser and Reese, 1981) and were mostly ~150 nm for dense-core vesicle exocytosis in *Limulus* amebocytes (Ornberg and Reese, 1981). In contrast, using cell-attached membrane capacitance recording, fusion pore conductance of secretory vesicles range from 30 to 1,000 pS (Breckenridge and Almers, 1987; Lindau and Alvarez de Toledo, 2003; He et al., 2006; MacDonald et al., 2006), corresponding to a fusion pore diameter of 1–7 nm. Fusion pores larger than 10 nm, as those observed under EM, will result in pore conductance approaching infinity, rendering estimation of pore size impossible. Additionally, these large fusion intermediates do not restrict the diffusion of neurotransmitters and small neuropeptides such as neuropeptide-Y (NPY) (Tsuboi et al., 2004). Thus, they are undetectable with either amperometry or membrane capacitance recordings. On the other hand, both electrophysiological methods provide an estimation of the pore duration before final dilation to be ~10–80 ms in endocrine cells on average. Therefore, it is intriguing that a small pore <10 nm was never observed under EM, provided that ultrafast-freezing EM should have sufficient temporal and spatial resolution in principal. Whether the small fusion pore intermediate is a rare event compared to other fusion intermediates or ultrafast-freezing EM lacks the resolution and contrast to resolve such small pores remains unknown. Live cell fluorescence microscopy, including super-resolution microscopy (Huang et al., 2009; Schermelleh et al., 2010), does not have the sufficient spatial and temporal resolution to observe small fusion pores either.

**Figure 1:**
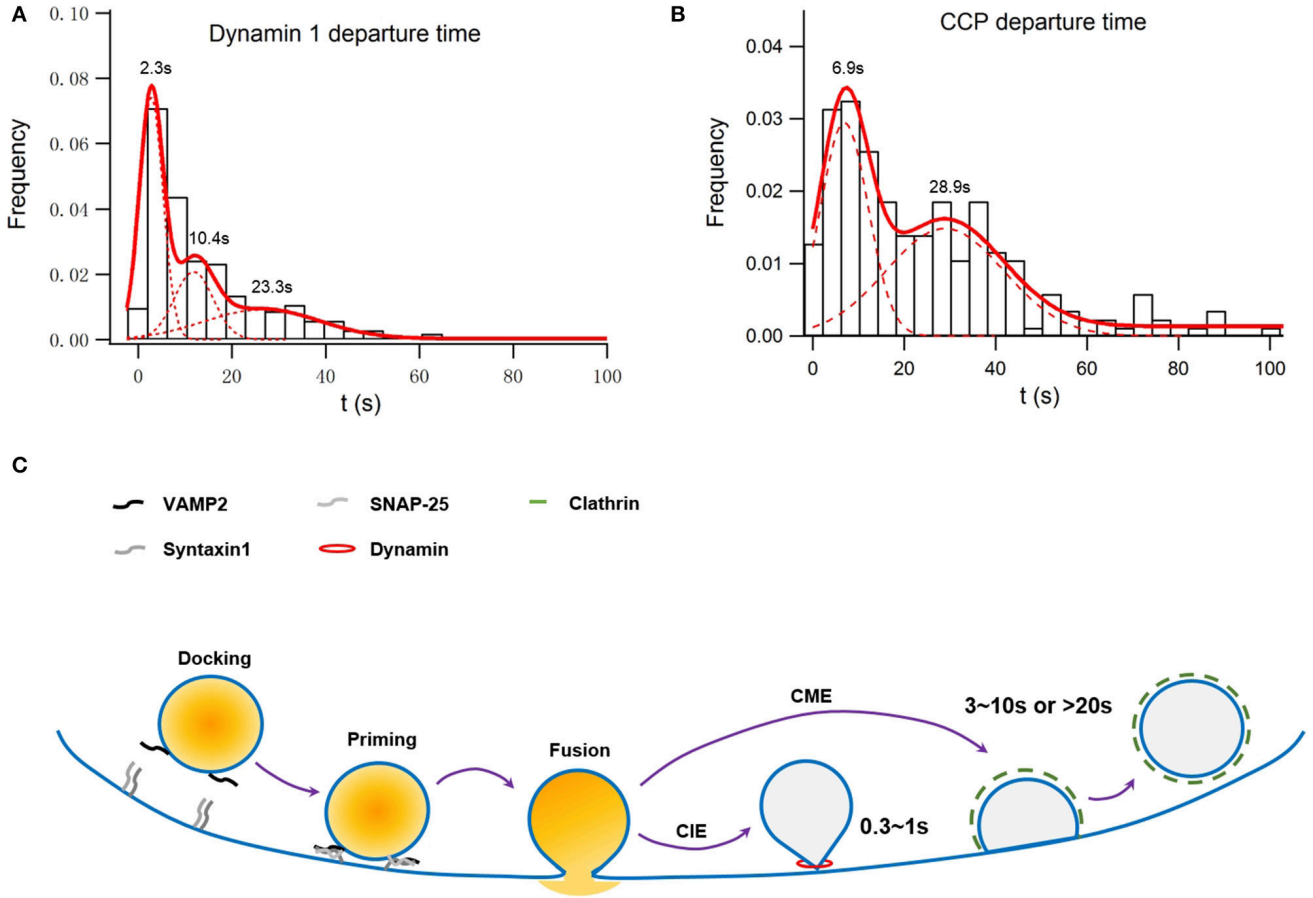
**Simulation of membrane capacitance decay at a fusion site due to clathrin-dependent endocytosis or both clathrin-dependent and -independent endocytosis**. INS-1 cells were transfected with VAMP2-pHluorin and clathrin-DsRed and were stimulated with 70 mM KCl and 15 mM glucose. Overall, 55 ± 3% of vesicle fusion events were associated with on-site recruitment of dynamin 1. **(A)** Normalized histogram of departure times of recruited Dyn1 puncta at fusion sites. It can be fitted with a three-component Gaussian distribution centered at 2.3 ± 0.11, 10.4 ± 0.5, and 23.3 ± 3.7 s and contributing to ~39 ± 3, 24 ± 6, and 34 ± 8% of the total population, respectively (*n* = 246). The latter two populations were dependent on clathrin, while the first one was independent of clathrin (Figure S2 in He et al., 2009). **(B)** Normalized histogram of departure times of recruited Clathrin puncta at fusion sites. It can be fitted with a two-component Gaussian distribution centered at 6.9 ± 0.5 and 28.9 ± 2.6 s and contributing to ~50 ± 4 and 44 ± 6% of the total population, respectively (*n* = 215). **(C)** A scheme of how clathrin-dependent and independent endocytosis are differently coupled to exocytosis.

The large fusion pore observed, on the other hand, may represent an intermediate before full collapse of vesicles. A secretory vesicle contains 60–70 copies of Syb2, of which 1–3 are used during the fusion process. All of these Syb2 molecules, as well as other vesicular proteins such as synaptotagmin, are completely lost on the plasma membrane during the full collapse of the vesicle. Clathrin-mediated endocytosis must be initiated to collect and precisely recycle these vesicular membrane components rapidly. Originally believed to be a slow process, we find that the migration of preformed clathrin-mediate pits (CCPs) on the plasma membrane to the vesicle release sites is key to the clearance of exocytic slots in a timely fashion (Figure 1; Yuan et al., 2015), which may explain the fast clathrin-mediated endocytosis observed in neurons (time constant of 3–10 s; Granseth et al., 2006; Zhu et al., 2009).

### Kiss and run (KR)

Resealing of a small fusion pore leads to a KR event, which is both an exocytic and an endocytic process. KR events are detected as the “stand-alone” foot signals in amperometry recordings (Zhou and Misler, 1996; Albillos et al., 1997) or membrane capacitance flickers in capacitance recordings (Albillos et al., 1997; He et al., 2006; MacDonald et al., 2006). However, electrophysiology methods cannot identify the integrity of the vesicular shape and composition after a vesicle performs a KR event. KR was originally defined by Ceccarelli et al. (1973) under EM as the fusion of vesicles with preservation of vesicle morphology. Therefore, the conservation of the vesicle shape, as observed with EM and live cell fluorescence microscopy, along with a small pore probed with electrophysiology technologies are cornerstone features of KR (Alabi and Tsien, 2013).

Various conditions have been shown to promote KR in a number of secretory cells, including high cytosolic Ca^2+^ (Ales et al., 1999) and activation of PKA (MacDonald et al., 2006). Despite this knowledge, the mechanisms of the resealing and flickering of a fusion pore remain elusive. In the lipidic pore model, each intermediate structure is at its free energy minima, and an injection of exogenous energy is needed for the transition between different fusion intermediates. Therefore, fusing vesicles remain connected to the plasma membrane with a narrow pore until the addition of new proteins and lipids to the fusion machinery reverses the process. Indeed, a variety of proteins, such as dynamin (Anantharam et al., 2011; Jackson et al., 2015), myosin II, actin (Aoki et al., 2010), SNARE proteins (Fang et al., 2008; Gucek et al., 2016), synaptotagmin (Wang et al., 2001; Lai et al., 2013), and complexin (Dhara et al., 2014) have been found to affect the fusion pore dynamics in chromaffin and PC12 cells. These studies highlight an active role of these components in impacting the fusion pore. Alternatively, it is hypothesized that the release of energy associated with the formation of one SNARE bundle is insufficient to overcome the restraining force from the intact vesicle-vesicle and vesicle-cytoskeleton filamentous web that opposes full vesicle collapse (Alabi and Tsien, 2013). Therefore, upon completion of the trans-SNARE complex formation and diminishing of the countering force against pore constriction, the vesicle pore reseals to be intact again. The difference between these two models is that the force opposing fusion dilation is constitutively present at the release sites in the latter model, therefore bypassing the need for acute and coordinated recruitment of facilitating membrane components.

The physiological significance of KR in endocrine cells has been well-established. In pancreatic β-cells, the KR of large dense-core vesicles (LDCV) and small vesicles allows for the selective release of ATP and GABA, respectively. In contrast, insulin crystals within LDCVs are retained within the lumen during the transient flickering of fusion pores (MacDonald et al., 2006). The release of peptides from other endocrine cells is also likely to be limited, since the transient brightening with no diffusion of fluorescent-tagged NPY puncta is regarded as a KR event under total internal reflection fluorescence (TIRF) microscopy (Tsuboi and Rutter, 2003). KR is also identified in the fusion of synaptic vesicles in synapses (He et al., 2006; Zhang et al., 2009), which may add another layer of post-fusional regulation and enables non-quantal synaptic transmission in principal. However, even for the smallest fusion pore opening, the vesicle will be drained of transmitter within tens of milliseconds, long before the fusion pore closes. Therefore, the KR model is unlikely to regulate vesicle release post-fusionally.

Alternatively, KR may confer an ultrafast and efficient recycling process independent of clathrin (He et al., 2006; Zhang et al., 2009). A KR event will lead to a fast and efficient recycling of almost all vesicular components, as well as immediate on-site refilling of neurotransmitters. The same vesicle then can fuse multiple times (Zhang et al., 2009), while the previously used cis-SNARE complex in the previous round of fusion needs to be removed from the vesicle to prevent blockade of the second round of fusion. However, it is unclear how such a cis-SNARE complex passes through the small flickering pore and diffuses into the plasma membrane without affecting the pore, given that the SNARE protein could be part of the pore itself. Nevertheless, by expelling a few Syb2 molecules used for each round of fusion, a KR event is more efficient in maintaining the identity of the vesicle than discharging all Syb2 molecules upon every instance of vesicle exocytosis. By keeping the vesicular V-ATPase, resealed vesicles can gradually re-acidify, which permits pH gradient-coupled refilling of the vesicle with neurotransmitters (Alabi and Tsien, 2013). In theory, this process may promote rapid recovery of neurotransmission during sequential stimulations. However, this hypothetical benefit is in disagreement with the experimental data that KR is prevalent at the beginning of action potential trains but is eventually replaced by full fusion upon sustained firing in hippocampal neurons (Zhang et al., 2009). Therefore, the physiological significance of KR in synapses remains unknown.

### Kiss and stay (KS)

In endocrine cells, in addition to probing fusion pores indirectly with electrophysiological methods, imaging technologies such as spinning disc confocal and TIRF microscopy provide the spatiotemporal correlation of fusion events with concurrent diffusion of vesicular lipids and proteins (Holroyd et al., 2002; Taraska et al., 2003; Tsuboi and Rutter, 2003; Tsuboi et al., 2004). In parallel to the small fusion pores detected from electrophysiological data, imaging reveals retention of vesicle membrane shape and some vesicle compositions after the exocytosis of LDCVs (Holroyd et al., 2002; Taraska et al., 2003; Tsuboi et al., 2004). In contrast to the retention of the majority of composition after a vesicle performs KR, the loss of vesicular lipids and the majority of some vesicular proteins such as Syb2 is obvious (Taraska and Almers, 2004; Tsuboi et al., 2004). Named as KS (or cavicapture), this process is regarded as an allosteric form of KR, sharing similar characteristics such as on-site recycling of vesicular components and its dependence on dynamin (An and Zenisek, 2004). In this sense, it is often regarded as a fast, clathrin-independent endocytosis that occurs at the fusion sites (Holroyd et al., 2002; Taraska et al., 2003; Tsuboi et al., 2004). However, we have shown that clathrin-dependent endocytosis could rapidly synchronized to occur at the fusion sites (Yuan et al., 2015), highlighting a necessity of classifying the identity of endocytosis based on molecules, rather than on kinetics and localization.

Moreover, it is unclear whether KS events identified by different fluorescence probes represent the same or different fusion intermediate stages. For example, discharge of NPY is much faster than that of fluorescent-tagged tissue plasminogen activator (tPA) in adrenal chromaffin cells and pancreatic β-cells (Tsuboi et al., 2004; Weiss et al., 2014). These data were initially interpreted to suggest that large tPAs (~10 nm in diameter) are prevented from free diffusion by the fusion pore, which do not interfere with diffusion of small lumen contents such as NPY (~3 nm in diameter). However, overexpressed tPA changes the lumen composition of LDCVs, binds to the exposed luminal surface of fused chromaffin granules and slows down the release kinetics as measured by amperometry recordings (Weiss et al., 2014). A pore as large as 10 nm barely constrains diffusion of small neurotransmitters from vesicle lumen upon exocytosis. Alternatively, KS may represent a fusion intermediate later than the KR, which explains why it shares many mechanistic characteristic with the later. Given the prevalence of KS in endocrine cells, we speculate that the 20–150 nm fusion pores observed under EM may be in the KS state, although future direct proof is still needed.

Clearly, resolving fusion-associated membrane shape retention and dispersion of vesicular membrane during synaptic transmission is difficult due to the small sizes of synaptic vesicles and boutons. However, coupled and recycled vesicular membrane proteins are distinct from those left on the presynaptic membrane after exocytosis (Wienisch and Klingauf, 2006), highlighting a possible KS mechanism operating in synapses as well.

### Sequential fusion and multivesicular exocytosis

After a KS fusion event, the invaginated fusion site before endocytosis can be targeted to harbor the next rounds of exocytosis (“sequential fusion”; Takahashi et al., 2004; Kishimoto et al., 2005), creating deep invaginations on the plasma membrane that may resulted in internalization of one large endocytic vesicle (“bulk endocytosis”; Wu and Wu, 2007; Wen et al., 2012). These invaginations were initially found in non-excitable cells such as pancreatic acinar cells, mast cells, eosinophils and neutrophils (Pickett and Edwardson, 2006) and were later discovered in excitable cells such as pancreatic β-cells and neurons (Kwan and Gaisano, 2005; He et al., 2009). During sequential fusion, cis-SNARE complexes need to be removed from the fusion sites, and new trans-SNAREs on the plasma membrane must diffuse into these invagination structures. By adopting this configuration, vesicles that exist deep within the cytosol readily fuse with the plasma membrane to release their contents. This configuration also creates spatially preferred sites on the plasma membrane, conferring a mechanism for generating exocytosis “hot spots” in non-neuronal cells.

Multivesicular exocytosis is another form of exocytosis where vesicles fuse homotypically before interacting with the plasma membrane. In contrast to compound fusion, a multivesicular exocytosis event leads to a capacitance increase that is several folds higher than that caused by the fusion of a single vesicle (He et al., 2009). However, multivesicular exocytosis is rare, precluding it from being systematically and statistically analyzed. Therefore, whether these large capacitance jumps represent a distribution different from that represented by a fusion of a single vesicle or the long-tail region of one unified distribution remains to be determined. Under EM, multiple vesicles that are connected to each other but not with the plasma membrane are sometime observed, which is also taken as evidence supporting multivesicular exocytosis (Wu L. G. et al., 2014). However, these structures could also be due to the sequential fusion of vesicles to the invaginated site that exhibited pore closure, which has a distinct molecular mechanism. A multivesicular exocytosis event needs homotypical vesicle-vesicle fusion, which presumably uses different sets of SNAREs other than those used for vesicle-plasma membrane exocytosis, similar to what has been proposed for compound fusion (Thorn and Gaisano, 2012). In principal, compared to the fusion of multiple vesicles at one designated site for several rounds, a multivesicular fusion event will be more efficient in emptying vesicular contents within a short period of time given that fusion sites are limited. However, how multivesicular fusion operates *in vivo* remains elusive.

## Coupled endocytosis in excitable cells

Unlike constitutive endocytosis in non-excitable cells, coupled endocytosis following evoked exocytosis must be fast and spatially matched with exocytosis to maintain the balance of surface membrane and the finite size of the readily releasable pool of vesicles. Kinetically, evoked endocytosis often consists of two phases, a fast endocytosis followed by a slow one (Artalejo et al., 2002; He et al., 2008; Lou et al., 2008; Wu et al., 2009). Mechanistically, the fast endocytosis is often regarded as clathrin-independent, while the slow one is often dependent on clathrin (He et al., 2008; Lou et al., 2008). Based on these kinetic and mechanical characteristics, a full collapsing of vesicle fusion is often thought to be associated with the slow, clathrin-dependent endocytosis, while the resealing of a fusion pore during a KR or KS is regarded as the fast mechanism underlying the coupled clathrin-independent endocytosis.

### Clathrin-dependent endocytosis

With immunostaining and confocal microscopy, active zones have been found to be surrounded by a peri-active zone enriched with endocytic proteins such as clathrin and dynamin, which mediate clathrin-mediated endocytosis following synaptic transmission (Cano and Tabares, 2016). Using TIRF microscopy, exocytosis in MIN6 cells was found to be associated with on-site recruitment of the endocytic protein dynamin but not clathrin, epsin, or amphiphysin. These data were interpreted to suggest that only clathrin-independent endocytosis, a form of KS, is spatially coupled to exocytosis in insulin-secreting β-cells (Tsuboi et al., 2004). However, do these spatially confined dynamin recruitments represent *bona fide* endocytosis? If they indeed represent clathrin-independent endocytosis, do their kinetics match with electrophysiological data? What is their physiological significance? These are questions left unexplored. Recently, we have systematically examined the exocytosis-endocytosis coupling in insulin-secreting cells (Yuan et al., 2015). We have revealed that clathrin can be recruited to the fusion sites in a fast and a slow manner, which were accompanied with the simultaneous recruitment of dynamin (Figures 1A,B refer to Figure S2 and Figure 1C in Yuan et al., 2015). The slow recruitment represents a *de novo* formation of clathrin-coated pits (CCPs), while the fast recruitment originates from preformed CCPs stably docked at the fusion sites or rapid movement of CCPs toward fusion sites on the plasma membrane. These spatially confined clathrin recruitments are indeed mediators of the endocytosis of vesicular proteins such as synaptotagmin VII and Syb2 (Yuan et al., 2015). Therefore, clathrin-dependent endocytosis can operate both at a fast and a slow pace, in agreement with similar findings in hippocampus neurons (Granseth et al., 2006; Zhu et al., 2009). We argue that the speed of designated endocytosis depends on the extent of synchronization of individual events, which cannot be used as the sole criteria for distinguishing clathrin-dependent from clathrin-independent endocytosis. As we have shown, the physiological significance of fast, clathrin-dependent endocytosis is also critical for sustained exocytosis during intense stimulation (Yuan et al., 2015), similar to what has been observed in synapses (Hosoi et al., 2009; Kawasaki et al., 2011).

### Clathrin-independent endocytosis

The recruitment of dynamin to fusion sites can be described by three Gaussian functions (Figure 1A refers to Figure S2 in Yuan et al., 2015). While the last two time constants match that of clathrin recruitment, the first one represents recruitment to sites ~2 s after a fusion event and independent of clathrin (Figure 1A). Assuming that dynamin 1-dependent endocytosis recycles a vesicle with a size similar to that of a dense-core granule (~230 nm in diameter) the membrane internalized by the clathrin-independent endocytosis shall be much larger than those internalized by the fast, clathrin-dependent pathway, in consistent of membrane capacitance experiments conducted in rat pancreatic β-cells (He et al., 2008). Such clathrin-independent fast recruitments of dynamin may also profoundly contribute to the fast membrane capacitance decay recorded in synapses and other endocrine cells (Artalejo et al., 2002; Lou et al., 2008; Hosoi et al., 2009). In addition to dynamin, actin also plays an indispensable role in the clathrin-independent endocytosis in pancreatic β-cells (He et al., 2008).

The identity of clathrin-independent, actin-dependent fast endocytosis is unlikely to be KR in β-cells, given that exocytosis with fusion pores smaller than 1 nm lasts <1 s in β-cells (Takahashi et al., 2002). Closure of a large fusion pore formed by KS (cavicapture) is likely to be the corresponding form of the fast clathrin-independent endocytosis. Bulk endocytosis, also found in endocrine cells and synapses (Wen et al., 2012; Watanabe et al., 2013), can be fast and independent of clathrin. Structurally, bulk endocytosis could be the reversal of sequential fusion or multivesicular endocytosis. The main difference between a bulk endocytosis and a cavicapture event is that the quantity of the plasma membrane retrieved by a single endocytic process is larger in the former. To differentiate these possibilities, we must directly visualize the membrane structures of fusion sites on the plasma membrane with imaging techniques. Of course, bulk endocytosis could also be unrelated to sequential exocytosis but related to a continuously invaginated plasma membrane driven by vesicular proteins, lipids, and endocytic machinery.

Finally, a clathrin-independent and dynamin-independent endocytosis is found in calyx neurons (Xu et al., 2008). However, without a definite molecular marker, this endocytic process cannot be studied further. In contrast, an ultrafast, clathrin-independent endocytosis is found in central synapses (Watanabe et al., 2013), which was inhibited by dynasore. However, given that dynasore affects cellular cholesterol, lipid rafts, and actin as well as dynamin (Preta et al., 2015), whether ultrafast endocytosis depends on dynamin remains to be proved. Actin is found to be required for the fast endocytosis in neurons (Delvendahl et al., 2016; Wu et al., 2016; Soykan et al., 2017), similar to what have been demonstrated in endocrine β-cells (He et al., 2008). However, how this fast endocytosis defined by electrophysiological and fluorescence experiments correlates with ultrafast endocytosis defined by the rapidly-freezing electron microscopy needs to be explored in the future.

## Molecular mechanisms for exo-endocytosis coupling

Different factors couple endocytosis with exocytosis, including cytosolic Ca^2+^, lipids, cytoskeleton and proteins (Wu L. G. et al., 2014). Here, we briefly summarize how they are proposed to function in exo-endocytosis coupling.

### Ca^2+^

Ca^2+^ influx through voltage-gated calcium channels triggers exocytosis in excitable cells. Synaptotagmin is the established Ca^2+^ sensor for triggering vesicle exocytosis. An increase in [Ca^2+^]_i_, on the other hand, accelerates but does not affect the amplitude of both clathrin-independent and clathrin-dependent endocytosis in β-cells (He et al., 2008). Not surprisingly, [Ca^2+^]_i_ elevation is found to initiate all forms of endocytosis (fast endocytosis, slow endocytosis, and bulk endocytosis) in calyx neurons (Hosoi et al., 2009; Wu et al., 2009). Because endocytosis is intimately linked to the prior exocytosis, the impact of Ca^2+^ influx on endocytosis may be a result of the impact of Ca^2+^ on membrane additions due to exocytosis. However, the relationship between the speed of endocytosis and [Ca^2+^]_i_ (He et al., 2008) is different than that between [Ca^2+^]_i_ and exocytosis (Wan et al., 2004). Similarly, deletion or mutation of both the C2A and C2B domains of the calcium-binding domains of synaptotagmin 1 prolongs the time constant of slow endocytosis by 30–50% but does not completely block the endocytosis (Yao et al., 2011). These data suggest that Ca^2+^ may affect the endocytic route via a pathway different from that for exocytosis.

The application of various calmodulin inhibitors blocks all types of endocytosis in calyx neurons, suggesting that calmodulin could be one Ca^2+^ sensor for endocytosis (Wu et al., 2009). The mechanism by which calmodulin phosphorylation initiates endocytosis remains to be determined. Calcineurin, the phosphatase that dephosphorylates many endocytic proteins (Cousin and Robinson, 2001), could be one main downstream target of calmodulin (Wu X. S. et al., 2014). Calcineurin has been shown to selectively dephosphorylate neuronal specific dynamin 1 and dynamin 3 but not ubiquitous dynamin 2. Such dephosphorylation is associated with the recruitment of F-BAR protein, syndapin I (Anggono et al., 2006), and may be critical for the stimulation of bulk endocytosis in synapses (Clayton et al., 2009). However, a large number of studies using blockers of calcineurin do not reach a consensus (Wu L. G. et al., 2014). Therefore, it is unclear whether such the controversy is due to the different synapses involved or a lack of specificity of pharmacological blockers. Resolving this issue is critical for understanding how calcium influx triggers endocytosis.

### Lipids

Phosphatidylinositol 4,5-bisphosphate (PIP_2_) is a minority phospholipid of the inner leaflet of plasma membranes (Suh and Hille, 2008). On the one hand, PIP_2_ activates voltage gated Ca^2+^ channels and slows channel rundown, which is upstream of vesicle exocytosis. On the other hand, PIP_2_ also interacts with a number of proteins essential for the exocytosis machinery, such as syntaxin 1, Munc13, synaptotagmin and Doc2, either via the C2 domain or via an electrostatic interaction with basic amino acids (Koch and Holt, 2012). PIP_2_ binds to syntaxin and Munc13, which regulate the readily releasable pool of vesicles, and the PIP_2_:synaptotagmin interaction seems to be essential for the Ca^2+^-dependent structure changes that catalyze the SNARE assembly. PIP_2_ also serves as a central hub for the organization of different endocytic proteins. Through electrostatic interactions with dynamin, the adaptor protein 2 (AP2), membrane curvature sensing protein FCHo, amphiphysin, and assessor proteins, such as epsin and synaptojanin, PIP_2_ facilitates the initiation, assembly, maturation, and final scission of CCPs. Therefore, PIP_2_, being in the center of recruiting proteins important for exocytosis and endocytosis, could be one crucial coupling factor.

Downstream of both Ca^2+^ and PIP_2_, we have shown that diaglycerol (DAG) could be another lipid that coordinates exocytosis and endocytosis. Ca^2+^ influx activates Ca^2+^-dependent phospholipase C, which breaks down PIP_2_ into inositol trisphosphate (IP_3_) and DAG, which is locally enriched around fusion sites in pancreatic β-cells. In return, DAG binds to Munc13 and activates protein kinase C, both of which are essential to vesicle exocytosis. As a lipid that induces negative membrane curvature, DAG microdomains accumulated at fusion sites reduce the energy of CCP movement on the plasma membrane, thus guiding the movement of preformed CCPs toward fusion sites to mediate fast, clathrin-dependent endocytosis (Yuan et al., 2015).

### Cytoskeleton

Densely packed actin filaments are often seen under the plasma membrane. Actin and related factors, such as Cdc42, N-WASP, and actin binding protein (ABP), interact directly or indirectly with active zone scaffolding proteins such as piccolo, organizing vesicle trafficking to, and fusion at the active zone. Cdc42 and N-WASP also interact with coat proteins of CCPs such as intersectin. These data suggest that actin could act as a bridge between exocytosis and endocytosis (Alabi and Tsien, 2013).

Microtubules, on the other hand, are often believed to bridge between the cell interior to the actin filaments close to the plasma membrane. However, microtubules originated from the Golgi can also touch the plasma membrane by CLASP, a microtubule-associated capping protein (Lansbergen et al., 2006). Through its interaction with LL5β, CLASP interacts with ELKS, another active zone scaffolding protein, and helps to anchor dynamic microtubule filaments at fusion sites. We show that a mutation of CLASP inhibits exocytosis in pancreatic β-cells and reduces coupled endocytosis along with a reduction in the simultaneous movement of CCPs toward the fusion sites (Yuan et al., 2015). Therefore, microtubules organized by CLASP and ELKS may be another factor that couples exocytosis with fast clathrin-dependent endocytosis in secretory cells.

### Proteins

SNARE proteins and associated proteins such as synaptotagmin and Munc13 are essential for exocytosis, while also interacting with proteins critical for endocytosis (Wu L. G. et al., 2014). However, different from their active roles in exocytosis, the roles of SNARE and associated proteins in endocytosis may be providing domains for AP2 and other adaptor proteins to recognize and bind. In this sense, their roles in endocytosis are permissive and non-essential. Dynamin is another protein that may participate critically in both exocytosis and endocytosis. As a GTPase, the role of dynamin in mediating fission of endocytic vesicle is well-known. On the exocytosis side, transfecting PC12 cells with a dynamin mutant with elevated GTPase activity shortened the foot duration of amperometry recordings, while the opposite occurred with the overexpression of a dynamin mutant with reduced GTPase activity (Anantharam et al., 2011; Jackson et al., 2015). These experiments place dynamin at the very beginning of exocytosis regulation, where the fusion pore is smaller than 1 nm. How this function of dynamin is correlated with its impact on the endocytic machinery remains elusive. Accordingly, deletion of dynamin-1 impairs both endocytosis and exocytosis at central synapses and produces different synaptic plasticity through distinct mechanisms (Mahapatra et al., 2016; Mahapatra and Lou, 2017); deletion of dynamin-2 in pancreatic β-cells leads to defects in clathrin-mediated endocytosis and biphasic insulin release (Fan et al., 2015).

## Summary and future perspectives

**Figure 2:**
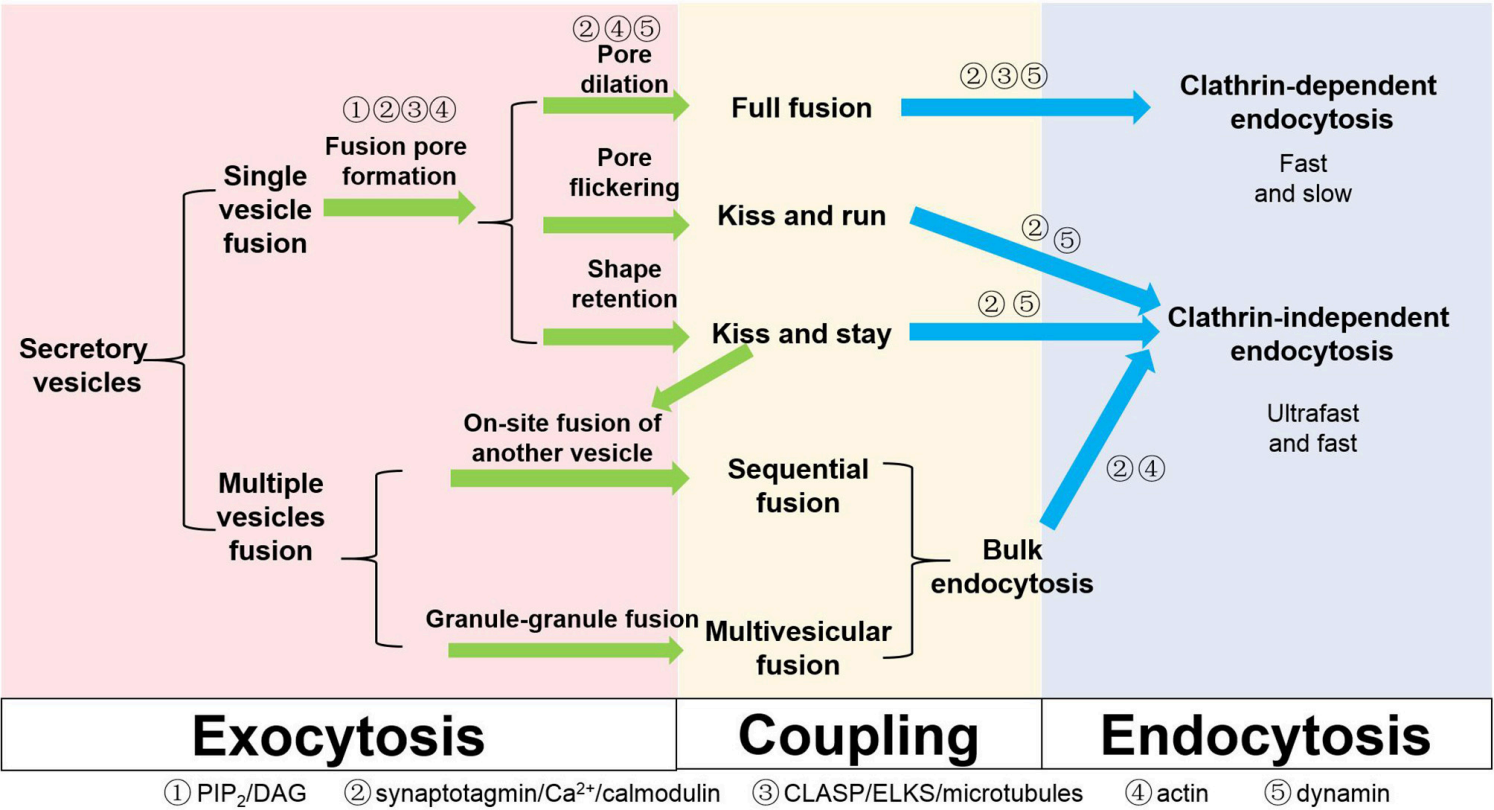
**Different types of exocytosis, endocytosis and coupling factors in secretory cells**. Coupling factors and their roles in different steps are also listed on the scheme.

We have summarized the above-mentioned mechanisms regarding exocytosis, endocytosis and possible coupling factors in Figure 2. From a macroscopic view, exocytosis may be matched with endocytosis: full fusion with clathrin-mediated endocytosis, KR and KS with clathrin-independent endocytosis, and sequential fusion and multivesicular exocytosis with bulk endocytosis. In this sense, the fate of the components of the fusing vesicle may be pre-determined at the moment of its choice of fusion modes. Therefore, understanding the early fusion intermediates of a vesicle, such as the hemifusion state, pore opening, dilation, and shape retention, will be instrumental for the understanding of the whole coupled process.

The listed classification of different exocytosis and endocytosis subtypes is not based on molecular mechanism but rather hinges on studies that involve different experiments conducted on different cell types. The terminologies defined by different methods may not be mutually inclusive or exclusive. For example, bulk endocytosis is usually regarded as a subcategory of clathrin-independent endocytosis. However, the bulk membrane invaginations observed in secretory cells under EM, which are often taken as evidence supporting bulk endocytosis, may support the internalization of small or large chunks of membrane in a clathrin-dependent manner in live cell studies. KR and KS may be one uniform process at different stages but could also be two distinct processes with non-overlapping mechanisms. To differentiate these controversies, it is important to sort out molecules that are exclusively used for some specific processes, in addition to actin for clathrin-independent endocytosis (He et al., 2008; Delvendahl et al., 2016). Alternatively, we shall examine the same process in the same cells using multiple techniques. For example, combining cell-attached membrane capacitance measurements with imaging vesicular lipids in endocrine cells will help clarify whether lipid exchange occurs between the vesicle and the plasma membrane during the flickering of a small fusion pore. Simultaneous imaging of vesicular components and extracellularly applied fluorescent dextran of different sizes will help monitor the dilation of a fusion pore from ~1 nm to a much larger in diameter (Takahashi et al., 2002). This will differentiate KR and KS and ultimately determine the size of fusion pores accompanying KS exocytosis. Monitoring the shape of the membrane may reveal clues of hemifusion in live cells (Zhao et al., 2016) and will also confirm or disapprove the compound fusion/multivesicular exocytosis theories and their physiological significance. Finally, operating at a nanometer scale with lifetimes of milliseconds, most of the fusion intermediate structures described here can hardly be directly discerned even with state-of-the-art super-resolution microscopy methodologies (Huang et al., 2009; Schermelleh et al., 2010). Despite differences in exocytosis kinetics and the organization of fusion sites between synapses and endocrine cells, we believe that the core exo-endocytosis coupling mechanism is conserved. Therefore, if we can improve the temporal and spatial resolution and duration of current super-resolution imaging technologies, direct visualization of fusion pore intermediates in endocrine cells may invoke new insights that would render much of the discussed theories here obsolete.

## Author contributions

All authors listed, have made substantial, direct and intellectual contribution to the work, and approved it for publication.

### Conflict of interest statement

The authors declare that the research was conducted in the absence of any commercial or financial relationships that could be construed as a potential conflict of interest.
